# Differential role of segments of α-mating factor secretion signal in *Pichia pastoris* towards granulocyte colony-stimulating factor emerging from a wild type or codon optimized copy of the gene

**DOI:** 10.1186/s12934-020-01460-8

**Published:** 2020-10-29

**Authors:** Sakshi Aggarwal, Saroj Mishra

**Affiliations:** Department of Biochemical Engineering and Biotechnology, Indian Institute of Technology Delhi, Hauz-Khas, New-Delhi, 110016 India

**Keywords:** Granulocyte colony-stimulating factor, *Pichia pastoris*, α-MAT secretory signal sequences, Recombinant therapeutics

## Abstract

**Background:**

The methylotrophic yeast, *Pichia pastoris* has been widely used for the production of human therapeutics, but production of granulocyte colony-stimulating factor (G-CSF) in this yeast is low.The work reported here aimed to improve the extracellular production of G-CSF by introducing mutations in the leader sequence and using a codon optimized copy of G-CSF. Bioinformatic analysis was carried out to propose an explanation for observed effect of mutations on extracellular G-CSF production.

**Results:**

Mutations in the pro-region of the α-mating type (MAT) secretory signal, when placed next to a codon optimized (CO)-*GCSF* copy, specifically, the Δ57–70 type, led to highest G-CSF titre of 39.4 ± 1.4 mg/L. The enhanced effect of this deletion was also observed when it preceded the WT copy of the gene. Deletion of the 30–43 amino acids in the pro-peptide, fused with the wild type (WT)-*GCSF* copy, completely diminished G-CSF secretion, while no effect was observed when this deletion was in front of the CO-*GCSF* construct. Also, Matα:Δ47–49 deletion preceding the WT-*GCSF* dampened the secretion of this protein, while no effect was seen when this deletion preceded the CO-*GCSF* copy of the gene. This indicated that faster rates of translation (as achieved through codon optimization) could overcome the control exercised by these segments. The loss of secretion occurring due to Δ30–43 in the WT-*GCSF* was partially restored (by 60%) when the Δ57–70 was added. The effect of Δ47–49 segment in the WT-*GCSF* could also be partially restored (by 60%) by addition of Δ57–70 indicating the importance of the 47–49 region. A stimulatory effect of Δ57–70 was confirmed in the double deletion (Matα:Δ57–70;47–49) construct preceding the CO-*GCSF*. Secondary and tertiary structures, when predicted using I-TASSER, allowed to understand the relationship between structural changes and their impact on G-CSF secretion. The Δ57–70 amino acids form a major part of 3rd alpha-helix in the pre-pro peptide and its distortion increased the flexibility of the loop, thereby promoting its interaction with the cargo protein. A minimum loop length was found to be necessary for secretion. The strict control in the process of secretion appeared to be overcome by changing the secondary structures in the signal peptides. Such fine tuning can allow enhanced secretion of other therapeutics in this expression system.

**Conclusions:**

Among the different truncations (Matα:Δ57–70, Matα:Δ47–49, Matα:Δ30–43, Matα:Δ57–70;30–43, Matα:Δ57–70;47–49) in pro-peptide of α-MAT secretion signal, Matα:Δ57–70 fused to CO-*GCSF*, led to highest G-CSF titre as compared to other Matα truncations. On the other hand, Matα:Δ30–43 and Matα:Δ47–49 fused to the WT-*GCSF* dampened the secretion of this protein indicating important role of these segments in the secretion of the cargo protein.

## Background

The advancements in genetic engineering technology have made possible to introduce novel recombinant drugs for the treatment of cancer, autoimmune diseases, diabetes, heart and genetic disorders [1, 2]. Being highly specific in action, these do not affect the natural biological processes. Recombinant human granulocyte colony-stimulating factor (hereafter referred to as G-CSF) is a 19.6 kDa glycosylated cytokine with proven efficacy against chemotherapy induced neutropenia [3, 4], for promotion of hematopoietic stem cell transplantation [5], in treatment of disorders of central nervous system [6, 7], in strengthening of the immune system of HIV patients [8] and for regeneration of heart tissues after myocardial infarction [9, 10]. Structurally, G-CSF is predominantly an α-helical protein, consisting of four antiparallel left-handed α-helices (A, B, C & D) with long interconnecting loops (AB, BC & CD) and a short E helix (3_10_ helix), located within the A–B loop with five cysteine residues at 17th, 36th, 42nd, 64th and 74th position [11, 12]. The natural G-CSF molecule is O-glycosylated at the Thr-133 position and while it enhances the circulatory half-life in blood, it is not necessary for its biological activity. Currently, three recombinant forms of G-CSF are available for therapeutic purpose, namely, Filgrastim and (Polyethylene Glycol) PEG-Filgrastim (that are *Escherichia coli* derived and non-glycosylated) and Lenograstim (Chinese hamster ovary cell (CHO) derived), this being expensive as compared to Filgrastim. The CHO derived product has not gained commercial importance as it carries the risk of being contaminated by viruses.

Apart from the bacterial platforms, a number of yeast species have been used for the production of human therapeutics. These include *Hansenula polymorpha, Kluyveromyces lactis, Pichia pastoris and Saccharomyces cerevisiae* [13, 14]*.* Among these, *P. pastoris* has been used most extensively for the production of recombinant therapeutics due to the presence of strong and tightly regulated Alcohol oxidase (*AOX1)* promotor [15], ability to achieve high cell densities [16–18], ability to carry out post-translational modification of proteins [19, 20] and easy downstream processing of the product [21–23]. Expression of heterologous proteins in the *P. pastoris* can be either intracellular or extracellular. Secretion of any protein into the extracellular medium requires the presence of a secretion signal sequence at the N terminus of the newly synthesized protein, which targets it to the secretory pathway. However, in some cases, the synthesized protein (also called as the cargo protein) fails to fold properly due to over-production/over-secretion and accumulates as misfolded protein. The accumulation of such misfolded proteins in the endoplasmic reticulum (ER) leads to protein aggregation, and, as a result, the expression of molecular chaperones and foldases is induced, thereby triggering the unfolded protein response (UPR) and ER-associated degradation (ERAD) pathways [24–26]. While several other leader sequences such as the *P. pastoris* acid phosphatase, PHA-E from *Phaseolus vulgaris* agglutinin, SUC2 from *S. cerevisiae* invertase [27–29], human serum albumin [30], the murine IgG1 signal peptide [31] have been used for the secreted expression in the *P. pastoris*, the α-mating type (α-MAT) secretion signal from the *S. cerevisiae* has been the most commonly and successfully used sequence.

**Fig. 1:**
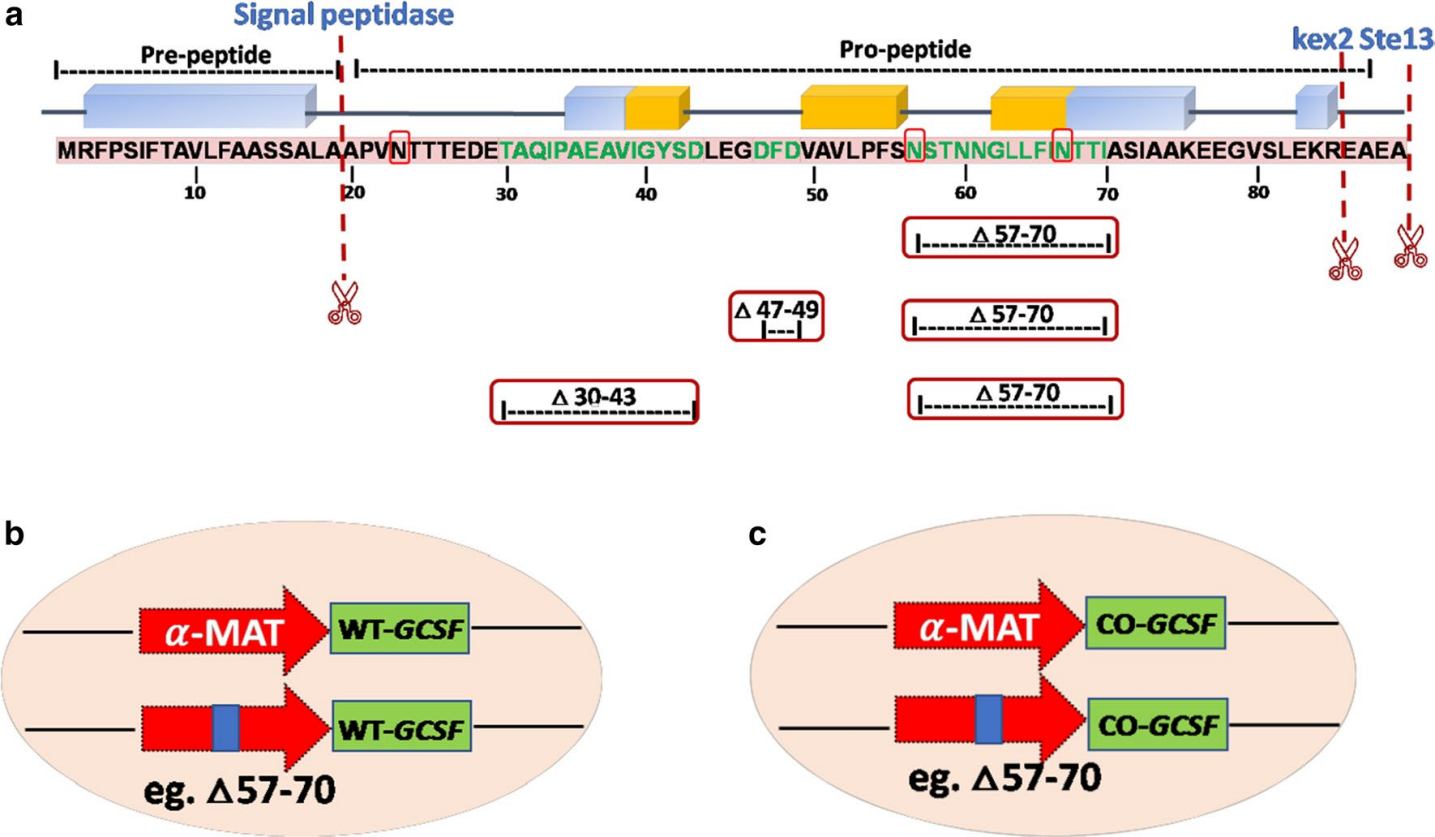
Schematic representation of truncations in the α-MAT secretory signal. **a** α-MAT consisting of the pre-region (19 amino acids), followed by a pro-region (66 amino acids) with three N-linked glycosylation sites (red box). Truncations, executed in the present study, in the pro-region of the α-MAT are shown. The position of the mutations is indicated by amino acid numbers **b** Schematic representation of the wild type and the truncated α-MAT (Δ57–70, shown as an example here) fused with the WT-*GCSF*. **c** Schematic representation of the wild type and the truncated α-MAT (Δ57–70, shown as an example here) fused with the CO-*GCSF*

The pre-pro- containing α-MAT leader sequence consists of a 19 amino acid (pre-) region followed by a 66 amino acid (pro-) region, containing three N-linked glycosylation sites and a dibasic (KR) kex2 endo-peptidase cleavage site (Fig. 1). The processing of the pre-pro signal peptide of the α-MAT occurs in three steps. First, the protein is recognized by SRP (signal recognition particle), which is involved in the translocation of cargo protein across the ER [32, 33]. Later, the pre-peptide leader sequence is cleaved off by the signal peptidase in the ER. At this point, N-glycosylation occurs at the three Asn residues in the pro-region of the α-MAT sequence. It is thought that this glycosylation may play a role in the facilitation of transport from ER to Golgi [34, 35]. Second processing occurs at the kex2 cleavage site, where the kex 2 endopeptidase cleaves off the peptide after the dibasic Lys-Arg residues. The pro-sequence consists of hydrophobic amino acids with short intermediate charged amino acids. The pro-peptide is thought to play a prominent role in protein folding and slows down the rate of transport in to the ER. Finally, the Ste13 peptidase cleaves off the Glu-Ala repeats in the pro-peptide of the secretory signal to generate mature α-factor peptide [36].

Previous studies have demonstrated that many heterologous proteins may not be secreted efficiently into the extracellular medium in the *P. pastoris* system. In spite of applying strategies, such as, codon optimization, increasing the copy number, combining both genetic and process conditions [37–39], overexpression of transcription factors [40], low production has marred the use of *P. pastoris* as an expression platform. Manipulation of the secretory signal sequences has shown promise for enhanced production of extracellular proteins in this yeast [41–44]. Production of G-CSF has been attempted in several strains of *P. pastoris* (X-33, SMD1168 and GS115) using the α-MAT secretory signal and reported yield varied from 2 to 20 mg/L of G-CSF at shake flask level [45–48]. Higher yields were reported by addition of Tweens which has not been confirmed by others. To the best of our knowledge, no systematic studies have been carried out to enhance the secretory efficiency of G-CSF by manipulation of the secretory signal sequences. The work reported here aimed to improve the extracellular production of human G-CSF by introducing mutations in the leader sequence. Bioinformatic analyses were carried out to understand how the individual and the combined truncations in the leader peptide affected the extracellular production of G-CSF. To understand the fine tuning between secretion and rates of translation, a native cDNA sequence (WT-*GCSF*) and a codon optimized synthetic gene (CO-*GCSF*) were taken as a system for investigation.

## Methods

### Strains, media, restriction and modifying enzymes

The *E. coli* DH5α strain was used for routine cloning, manipulation and transformation experiments. *P. pastoris* X-33 strain (Invitrogen, USA) was used for expression of G-CSF. The WT-*GCSF* (native cDNA encoding human G-CSF), cloned in pUC57 vector, was obtained commercially (Sigma-Aldrich). The *E. coli* DH5α and *P. pastoris* X-33 were maintained on Luria-Broth (LB) and yeast-extract, peptone, dextrose (YPD) medium (containing 20% glycerol) respectively and stored at − 80 °C. The media used and their composition was as per standard protocols [49**,** *Pichia* Easy Select Lab Manual]. The *E. coli*-*P. pastoris* shuttle vector pPICZαB (Invitrogen, Carlsbad, CA, USA) was used for cloning and expression studies. The *E. coli* cells, containing the recombinant vector, were grown in LB containing 25 μg/ml zeocin (Life Technologies, Carlsbad, CA). *P. pastoris* was cultivated in YPD medium while the recombinants were grown in YPD containing zeocin (100 μg/ml). Restriction endonucleases (*Xho*I*, Xba*I*, Sac*I*, Dpn*I) were procured from New England Biolabs (NEB). RNase used in regular DNA isolation experiments was purchased from GeneDirex. T4 DNA ligase (Thermo Fisher Scientific) was used for ligation reactions during cloning experiments. Plasmid isolation kit, gel extraction kit and PCR purification kit were purchased from Qiagen, USA. DNA ladder (1 Kb) and Blue-Ray pre-stained protein molecular weight ladder were purchased from GeneDirex. Zeocin, a selectable marker was purchased from Invitrogen. Taq DNA polymerase, used for routine PCR reactions, was purchased from Kapa Biosystems. High fidelity Phusion polymerase was purchased from Thermo Fisher Scientific.

### Construction of plasmids

**Fig. 2:**
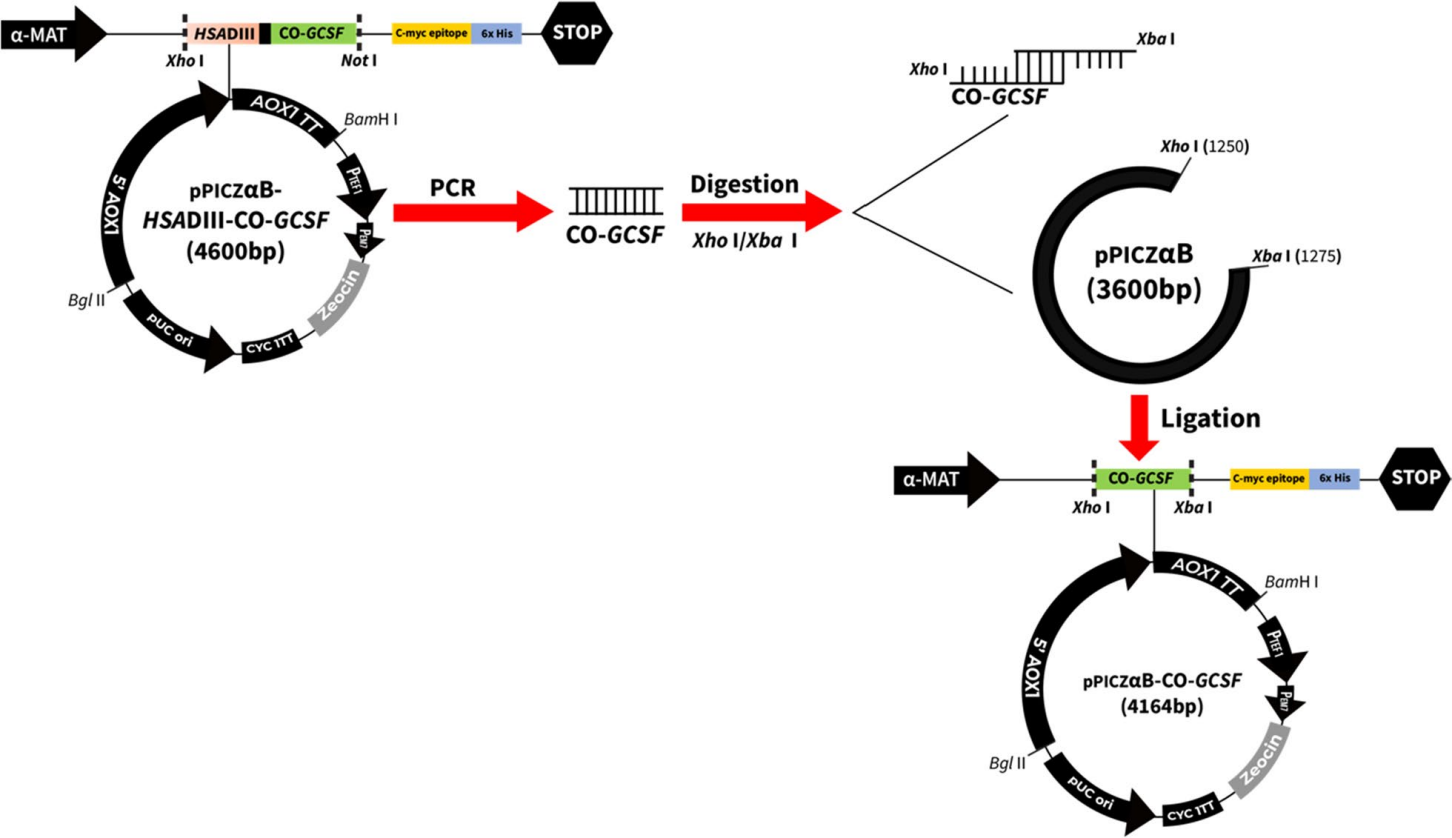
Scheme for construction of the plasmid containing the CO-*GCSF* gene. A copy of the CO-*GCSF* gene was amplified from pPICZαB-*HSA*DIII-CO-*GCSF* construct [50] using *G-CSF* specific forward and reverse primers containing appropriate restriction sites. The restriction enzyme digested (*Xho* I and *Xba* I) vector and the amplified *G-CSF* fragment were isolated from the gel, purified and ligated to form the pPICZαB-CO-*GCSF *

Yeast expression vector pPICZαB was utilized as a parent plasmid for construction of various mutations in the α-MAT secretory signal sequence. In order to construct the plasmid pPICZαB-CO-*GCSF*, containing the codon optimized copy of the gene [48], forward primer (5′CATCTCGAGAAAAGAGAGGCTGAAGCTACCCC3′) and a reverse primer (5′ATCTCTAGACTATTAGGGCTGGGCCAAGTGTCTCAAAAC3′) were utilized to amplify out the CO-*GCSF* gene from a fusion construct, reported previously [50]. The fusion construct was in the plasmid pPICZαB-*HSA*DIII-CO-*GCSF*, containing a gene fragment, encoding Domain III of human serum albumin (HSA) and CO*-GCSF* copy of the gene (Fig. 2). The purity/length of the amplified product (CO-*GCSF* with the flanking *Xho*I and *Xba*I sites) was checked on 1% agarose gel. Amplified product and the vector were double digested with *Xho*I/*Xba*I separately, cleaned and ligated using T4 DNA ligase. Transformation was carried out in *E. coli* as per standard methods [49] and recombinants selected on LB + zeocin as described above. The propagation and large-scale preparation of the recombinant vector was carried out using *E. coli*. The recombinant vector was sequenced using CO-*GCSF* specific primers to confirm the correctness of the reading frame.

In a similar manner, the commercially obtained pUC57 vector, containing the native human cDNA of G-CSF (WT-*GCSF*), was processed using restriction enzymes and cloned in the vector pPICZαB, downstream of the α-MAT secretory signal sequence and the construct called WT-*GCSF*.

### Truncations in the pro-secretory sequences

Deletion mutagenesis was carried out using Quick-change II XL Site-Directed Mutagenesis Kit from Stratagene (La Jolla, CA, USA). The HPLC purified primers used in this study are listed in Table 1. These were diluted to 50 ng/μl. The reaction mixture containing, 5 μl of 10 × reaction buffer, 1 μl parent plasmid, 10 μl of each primer, 1 μl of 10 mM dNTP, 3 μl of Quick-solution, 1 μl *Pfu*Turbo DNA polymerase, was made to a final volume of 50 μl with sterile distilled water. Cycling parameters for the PCR reaction were: 1 cycle of 95 °C for 5 min; 18 cycles of 95 °C for 30 s, 55 °C for 1 min, 68 °C for 7 min, followed by a single cycle of 68 °C for 10 min. An aliquot of 5 μl of the reaction mixture was run on 1% agarose gel to confirm amplification. The amplified PCR product was digested with 1 μl of *Dpn*I enzyme and incubated for 2 h at 37 °C, followed by heat inactivation of the enzyme at 80 °C for 20 min. Finally, the digested product was purified using the Qiagen PCR purification kit. Ten μl of the purified product was transformed into DH5α chemically competent cells and the mixture was plated on LB agar medium containing 50 μg/ml zeocin and incubated overnight at 37 °C. A few of the transformants were analysed for the presence of the plasmid by colony PCR method. One of the positive transformants, from each category of the mutants, was cultivated on large scale, for preparation of the plasmid for electroporation in to *P. pastoris*. The WT-*GCSF* and the CO-*GCSF* constructs, containing the native pro-region of the α-MAT signal sequence, served as the control.

**Table 1:** Mutagenic primers used to carry out truncations in the α-MAT secretory signal

Primers	Primer Sequence
Matα:WT	GCTGTTTTGCCATTTTCCAACAGCACAAATAACGGGTTATTGTTTATAAATACTACTATTGCCAGCATTGCTGCTAAAG
Matα:Δ57–70	F.P: 5′-GCTGTTTTGCCATTTTCCGCCAGCATTGCTGCTAAAG-3’ R.P: 5′-CTTTAGCAGCAATGCTGGCGGAAAATGGCAAAACAGC-3’
Matα:WT	CTACAACAGAAGATGAAACGGCACAAATTCCGGCTGAAGCTGTCATCGGTTACTCAGATTTAGAAGGGGATTTCG
Matα:Δ30–43	F.P: 5′-CTACAACAGAAGATGAATTAGAAGGGGATTTCG-3’ R.P: 5′-CGAAATCCCCTTCTAATTCATCTTCTGTTGTAG-3’
Matα:WT	CGGTTACTCAGATTTAGAAGGGGATTTCGATGTTGCTGTTTTGCCATTTTCC
Matα:Δ47–49	F.P: 5′-CGGTTACTCAGATTTAGAAGGGGTTGCTGTTTTGCCATTTTCC-3’ R.P: 5′-GGAAAATGGCAAAACAGCAACCCCTTCTAAATCTGAGTAACCG-3’

The underlined sequences were removed in the different α-MAT deletion constructs. The corresponding WT α-MAT sequences are shown above the deletion

### Transformation of *P. pastoris* with plasmid DNA containing confirmed mutations in the pro-region of the secretory signal and establishing their methanol utilisation pattern

For high-efficiency electroporation, around 30–50 ng of plasmid, from each mutant and wild type construct, was linearized using *Sac*I enzyme and the digestion of circular plasmid was checked by loading samples on 1% agarose gel. X-33 electrocompetent cells (40 μl) were mixed with 30 ng of linearized DNA in 0.2 cm electroporation cuvette (Bio-Rad). Electroporating pulse was applied at 1.5 kV, 25 μF, 186 Ω using Bio-Rad electroporator and 1 ml of ice-cold 1 M sorbitol was added in each cuvette. The cuvettes were incubated for 2 h at 30 °C in static condition. About 50–200 μl of cells was plated on YPDSA medium containing zeocin at 100 μg/ml. The plates were monitored for 3–4 days to check for the appearance of the transformed colonies.

The genomic DNA was isolated by glass beads method as described previously [51]. The screening of the transformants, containing the integrated gene (WT-*GCSF* or the CO-*GCSF*) was carried out by PCR method using gene specific primers. The isolated DNA was used as a template after dilution (1:20) and the PCR was performed using the following conditions: initial denaturation at 95 °C for 5 min, followed by 32 cycles of the following runs (denaturation at 95 °C for 30 s, annealing at 58 °C for 30 s and elongation at 72 °C for 50 s) and final elongation at 72 °C for 10 min. The amplified product was analysed by agarose gel electrophoresis. The positive transformants from each category of mutants were also screened for methanol utilization phenotype (Mut). For this, the PCR reaction was set up using universal alcohol oxidase (*AOX1*) primers and the amplified product was checked on 1% agarose gel. PCR cycle was carried out as follows: Initial denaturation at 95 °C for 5 min, 32 cycles of the following runs (denaturation at 95 °C for 30 s, annealing at 55 °C for 30 s, elongation at 72 °C for 2 min) and final elongation at 72 °C for 10 min.

### High throughput screening of the *P. pastoris* transformants for the production of G-CSF in a 48-well plate

Around 15 transformants from each mutant category were cultured in triplicates. The colonies were revived on YPDA (1% yeast, 2% peptone, 2% dextrose, 2% agar) for 48 h at 30 °C. An overnight culture of the same was prepared in the YPD medium. This was used to inoculate directly 500 μl of buffered complex methanol medium (BMMY: 1% yeast extract, 2% peptone, 100 mM potassium phosphate, pH 6.0, 1.34% yeast nitrogen base, 4 × 10^−5^% biotin and 0.5% methanol) and the culture incubated for a total period of 72 h at 28 °C with shaking at 220 rpm. Induction of G-CSF was carried out by addition of 1% methanol every 24 h. Final cell O.D at 600 nm, total extracellular protein and pH were monitored for all the transformants. Equal volume of culture supernatant was loaded on SDS-PAGE and the gel was stained by silver staining (see Analytical methods). G-CSF and total extracellular protein production were monitored by gel densitometry and Bradford reagent respectively. For every category of mutant, suitable control (transformant containing the plasmid with either the WT-*GCSF* copy or the CO-*GCSF* copy preceded by the native pro-region of the α-MAT signal sequence) were run in parallel in every experiment.

### Methanol inducible production of G-CSF by *P. pastoris* transformants at shake flask level

Two representative clones were selected from each category of mutants (one set comprising of different mutants in the α-MAT pro-secretory signal preceding the WT-*GCSF* copy and the other comprising of different mutants in the α-MAT pro-secretory signal preceding the CO-*GCSF* copy). Production of G-CSF was monitored in parallel in each set to avoid non-uniform cultivation conditions, as different batches were screened in different 48-well plates. A list of the mutants is given in Table 2. For this, a single colony was inoculated into 20 ml of YPD medium containing 100 μg/ml of zeocin and cultivated overnight at 30 °C, 220 rpm. A small aliquot of cells was transferred into buffered complex glycerol medium (BMGY: same as the BMMY except that 1.0% glycerol replaced 0.5% methanol) to set the initial O.D. at 0.2. The culture was grown in a shaking incubator at 28 °C, 220 rpm to an O.D. of 2–6 (~ 16–18 h). The cells were harvested by centrifugation at 6000*g* for 10 min. The cells were next transferred to 100 ml of BMMY medium (contained in 500 ml flask) and induced with 1% methanol every 24 h for G-CSF production. Incubation was carried out at 28 °C, 220 rpm for 120 h. Cell OD at 600 nm, pH and total extracellular protein was monitored every 24 h. After harvesting, clear culture supernatant was analysed on SDS-PAGE. Equal volume (10 μl) of the culture supernatant was loaded for analysis of extracellular protein and the G-CSF produced. The protein bands were visualized after silver staining.

**Table 2:** List of clones selected from each category of mutants

Mutations	WT-*GCSF*	CO-*GCSF*
Matα:WT	2	6, 10
Matα:Δ57–70	6, 16	25, 32
Matα:Δ30–43	10, 16	12, 32
Matα:Δ47–49	24, 30	44, 45
Matα:Δ57–70;30–43	21, 34	23, 33
Matα:Δ57–70;47–49	22, 25	17, 18

## Bioinformatic analyses

The nucleotide sequence of the wild type and the mutated α-MAT secretory signal were retrieved from the raw chromatogram using Snap Gene viewer and the corresponding amino acid sequence obtained from NCBI. Molecular modelling of the pre-pro α-MAT was done using I-TASSER. Secondary structures and 3-D models were generated for the native and the mutated α-MAT secretory signal sequences using I-TASSER [52, 53].

## Analytical methods

The cell growth was monitored by measuring O.D at 600 nm every 24 h using visible spectrophotometry (Perkin Elmer UV–Vis spectrophotometer). If the O.D. of the culture was more than 0.9, the samples were diluted with water to attain the desirable range. Dry cell weight measurements were also carried out to link it with cell O.D. Total extracellular protein in the culture supernatant was measured by the Bradford assay using Quick Start™ Bradford 1 × reagent (Bio-Rad).

The cell-free culture filtrate, obtained after 120 h of cultivation, was filtered through a 0.2 μm filter (Axiva Sichem biotech). The filtrate was checked for production of G-CSF by SDS-PAGE on a 12% resolving gel. Equal volume (25 μl from the 48-well plate grown culture or 10 μl from the 100 ml grown culture) of the culture supernatant (from each category of mutants) was mixed with 4× SDS loading buffer and samples boiled at 95 °C for 10 min. This was followed by centrifugation at 15000*g* for 5 min. In parallel, G-CSF standard (Filgrastim at different concentrations) was loaded for verification and quantification of the G-CSF. Tris–glycine buffer (1×) with 0.1% SDS was used as a running buffer. Initially, the gel was run at 10 mA until it crossed the stacking gel followed by running it at 20 mA in the resolving gel. The gels were silver stained after fixing them and washing thoroughly as per standard protocols [54] and the amount of secreted G-CSF quantified based on the standard prepared with gel densitometry.

The G-CSF bands obtained under different experimental conditions were quantified using the gel densitometric method described previously [48]. Gel doc XR- Imaging system (Bio-Rad) was used for gel scans and the bands were quantitated using 1D Analysis software, Quantity One (Bio-Rad). Commercially available Filgrastim (Grafeel®, Dr. Reddy’s Laboratories Ltd., India) was used as a standard reference in the linear range of 0.3–4.5 μg/ band. Gaussian trace quantity (Intensity x mm) was calculated for each band and compared with the standard reference (Filgrastim) for quantification of G-CSF.

The identity of the protein band obtained at the expected ~ 19 kDa position (in the SDS-PAGE under the electrophoretic conditions used) was established by MALDI-TOF (Matrix Assisted Laser Desorption/ Ionization- Time of Flight). For this, the total proteins were precipitated from the culture supernatant (obtained from cultivation of *P. pastoris* transformants containing either the WT-*GCSF* or the CO-*GCSF* construct) obtained at 120 h, using previously described 12% TCA-acetone precipitation method [55]. The concentrated protein sample (20 μl) was loaded in 5 lanes of 12% SDS-PAGE and resolved. The gel was stained using Coomassie brilliant blue. The stained G-CSF band was excised with the help of sterile scalpel and stored in sterile Eppendorf tubes. Precautions were taken to avoid keratin contamination in the SDS gel. The sample was then sent to commercial service provider (Chromous Biotech, India) for in-gel tryptic digestion and peptide mass finger printing by MALDI-TOF/MS.

## Results

### Cloning of the WT-*GCSF* and the CO-*GCSF* in pPICZαB vector and verification of deletion mutations in the pro-segment of the α-MAT signal sequence through sequencing

The WT-*GCSF* pPICZαB construct containing the cDNA copy of the human G-CSF (from recombinant pUC57) was sequenced to confirm the correctness of the reading frame. A copy of the CO-*GCSF* gene, amplified from the *HSA*DIII-CO-*GCSF* construct [50], was also successfully cloned in the pPICZαB and the construct sequenced to determine the correctness of the reading frame. As reported earlier [50], the fusion construct lead to production of increased levels of the hybrid protein but due to the presence of additional sequences, those of the Domain III of HSA, cannot be used for applications requiring only G-CSF. Both the plasmid constructs were passaged through *E. coli* and then stably maintained in the *Pichia* transformants.

Truncations were carried out in the native α-MAT pro-secretory signal sequence (Fig. 1a), preceding the (i) WT-*GCSF* gene (Fig. 1b) or (ii) the CO-*GCSF* gene (Fig. 1c). The native α-MAT signal sequence preceding the WT-*GCSF* or the CO-*GCSF* gene served as the corresponding control. The colonies that appeared after the transformation of the mutant constructs were screened for the presence of the *GCSF* gene. Amplification of a DNA band at ~ 564 bp (as predicted) confirmed the positive recombinants. Two colonies from each category of mutants were sequenced and the results confirmed the desired deletions in the α-MAT signal peptide. The *Pichia* transformants obtained from each category of mutants were checked for the methanol utilization (Mut) phenotype. In transformants containing the MATα:Δ57–70 deletion, amplification of two bands, the upper band (2.2 kb) and the lower band (1.2 kb), confirmed that the transformants were of the Mut^+^ phenotype (Additional file 1: Fig. S1). The other mutant constructs (MATα:Δ30–43; MATα:Δ47–49, MATα:Δ57–70;30–43, MATα:Δ57–70;47–49) were also concluded to be of the Mut^+^ type based on the amplification of two bands.

### High throughput screening of the *P. pastoris* transformants for production of extracellular G-CSF with truncated pro-secretory sequences preceding the WT-*GCSF* gene

**Fig. 3:**
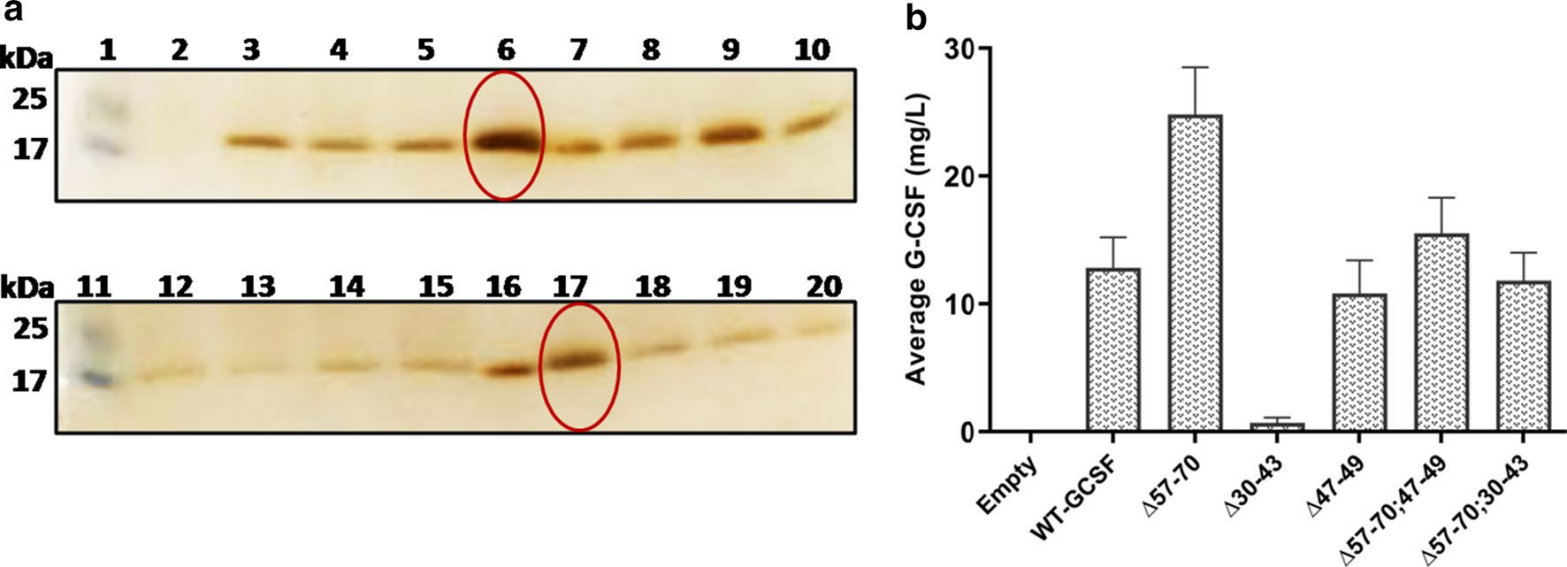
**a** SDS-PAGE analysis of extracellular G-CSF produced in BMMY medium by the truncated Matα fused to the WT-*GCSF* gene. Equal volume (25 μl) of the culture filtrate was loaded after 72 h of cultivation in a 48-well plate. Representative image of Matα:Δ57–70 transformants is shown. Lanes 1 & 11: Mol wt markers; Lane 2: empty vector; Lanes 3, 4, 5, 6, 8, 9, 10: Cl #s 3 to 9; Lane 12: Matα:WT; Lanes 13, 14, 15, 17, 18, 19, 20: Cl #s 13 to 19; Lanes 7 & 16: Standard Filgrastim (2.0 μg & 2.5 μg respectively)  **b** Average extracellular G-CSF (mg/L) production from 14/15 clones of each category at microplate level 72 h post-methanol induction

Out of a large number of transformants obtained from single deletion mutants, around 30 transformants were selected from each category (Matα:Δ57–70; Matα:Δ47–49; Matα:Δ30–43) and cultivated directly in the BMMY medium in a 48-well plate. The data for 30 such clones was collected. A representative gel image is shown for 14 transformants from the Matα:Δ57–70 category (Fig. 3a) and the remaining 16 clones are shown in Additional file 1: Fig. S2A. As seen, considerable variation was obtained in the level of extracellular G-CSF among the different clones, as has been widely reported earlier [48, 56–59]. Based on the gel densitometry data, average value for G-CSF production was determined from the 15 clones, screened under each category of mutants, and the data are shown in Fig. 3b. As seen, maximum extracellular production of ~ 25 mg/L was obtained in the Matα:Δ57–70 deletion group which represented a nearly 2-fold increase in extracellular G-CSF over that produced from the WT α-MAT signal. Deletion in the amino acids 30–43 (Δ30–43) led to failure of detection of G-CSF in the extracellular culture filtrate indicating an importance of this segment in the secretion process. However, the effect of this deletion was compensated for by the Δ57–70 deletion (Fig. 3b). Similarly, deletion in the amino acids 47–49 segment led to a slight decrease in extracellular G-CSF which was again compensated for and stimulated by deletion in the 57–70 region. Among the Matα:Δ57–70 clones, Cl #s 6 and 16 were selected for further evaluation. Similarly, representatives from each category of mutants were selected, the list of which is given in Table 2. All these were evaluated at shake flask level for extracellular production of G-CSF.

### Comparative analysis of G-CSF production among *P. pastoris* transformants containing different truncated pro-secretory sequences fused to the WT-*GCSF*

**Fig. 4:**
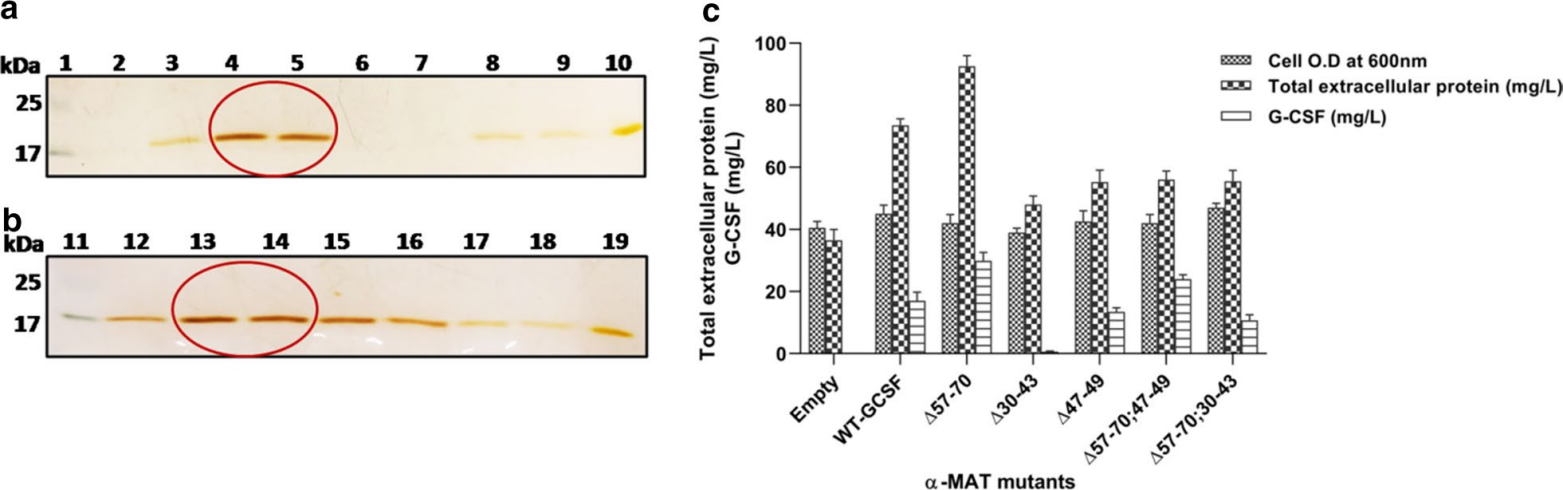
**a** SDS-PAGE analysis of the extracellular G-CSF produced by transformants containing single deletion in the pro-secretory sequence fused to the WT-*GCSF* gene. Equal volume (10 μl) of the culture filtrate was loaded after 120 h of cultivation in a 500 ml Erlenmeyer flask. Lane 1: Molecular weight ladder; Lane 2; empty vector; Lane 3: Matα:WT fused to the WT-*GCSF* gene; Lanes 4 & 5: Matα:Δ57–70, Cl #s 6 & 16; Lanes 6 & 7: Matα:Δ30–43, Cl #s 10 &16; Lanes 8 & 9: Matα:Δ47–49, Cl #s 24, 36; Lane 10: Filgrastim as a standard (1.5 μg). **b** SDS-PAGE analysis of G-CSF produced by transformants containing double deletions in the pro-secretory sequence fused to the WT-*GCSF* gene. Lane 11: Molecular weight ladder; Lane 12: MATα:WT fused to the WT-*GCSF* gene; Lanes 13 &14: Matα:Δ57–70, Cl #s 6, 16; Lanes 15 &16: Matα:Δ57–70;47–49, Cl #s 22, 25; Lanes 17 & 18: Matα:Δ57–70;30–43, Cl #s 21, 34; Lane 19: Filgrastim (1.5 μg). **c** Cell O.D, total extracellular protein and G-CSF titre at 120 h post-methanol induction. The units for cell O.D. are the same as shown on the Y-axis

The selected producer clones were analysed at shake flask level, under uniform conditions of cultivation, for extracellular production of G-CSF (Fig. 4a, b). While primary screening can be carried out at 48-well plate, aeration, maintenance of uniform pH and temperature, may not be possible under these conditions, and, as these affect cell density and thereby yield of the foreign protein, the mutants need to be evaluated under standard cultivation conditions. The results confirmed the findings obtained earlier (see the preceding paragraph) and the details are provided below. Among the transformants, the cell O.D was found to be between 40 and 48 with the final pH value of 6.0–6.2 in all the transformants. Total extracellular protein varied from 50 to 90 mg/L among different transformants (Fig. 4c). Deletion of the 57–70 amino acid residues in the pro-peptide resulted in a ~ 1.8-fold increase in G-CSF level to 29.8 mg/L when compared to that obtained with the native pro-peptide signal (~ 17 mg/L). Deletion in the 30–43 amino acids abolished extracellular production of G-CSF and when this was coupled with the deletion of 57–70 amino acids, the secretion of G-CSF was restored by about 60% with respect to the wild type α-MAT fused with the WT-*GCSF*. This implied that 30–43 are important residues of the pro-secretory signal and play a crucial role in secretion of G-CSF. Also, Matα:Δ47–49 led to production of ~ 13.5 mg/L of G-CSF which was slightly less than that obtained with the native leader sequence indicating the importance of this section. Again, when the deletion of 47–49 amino acids was coupled with Matα:Δ57–70, the secretory efficiency of G-CSF was partially restored but was not as high as when Δ57–70 occurred singly. A summary of these results is presented in Fig. 4c.

### High throughput screening of the *P. pastoris* transformants for production of extracellular G-CSF with truncated pro-secretory sequences preceding the CO-*GCSF* gene

**Fig. 5:**
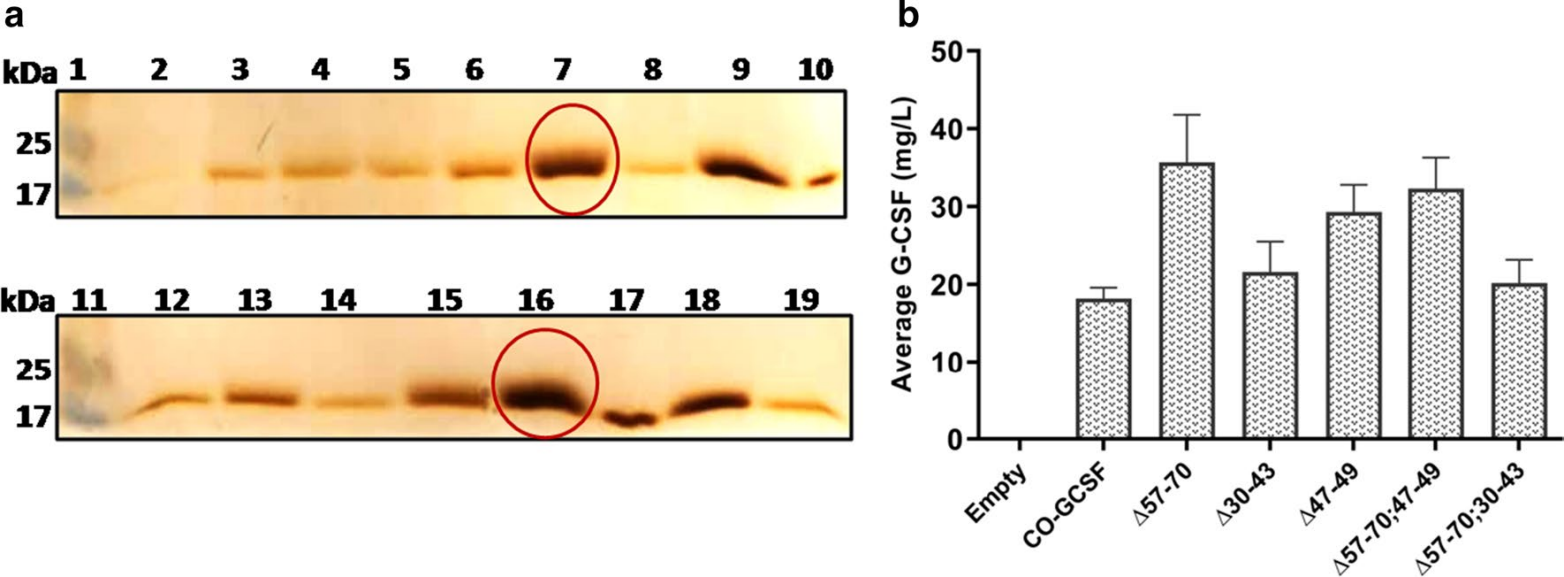
**a** SDS-PAGE analysis of the extracellular G-CSF produced in BMMY medium by the truncated Matα fused to the CO-*GCSF*. Equal volume (25 μl) of the culture filtrate was loaded after 72 h of cultivation in a 48-well plate. Representative image of Matα:Δ57–70 transformants is shown. Lanes 1 & 11: Mol wt markers; Lane 2: empty vector; Lane 3: Matα:WT fused to the CO-*GCSF* gene; Lanes 4 to 9: Cl #s 22, 23, 24, 25, 26, 27; Lanes 10 & 17: Standard Filgrastim (1.5 μg and 4.5 μg respectively); Lanes 12 to 19: Cl #s 28, 29, 30, 31, 32, 34, 35. **b** Average extracellular G-CSF (mg/L) production from 13 clones of each category at microplate level, 72 h post-methanol induction

Out of a large number of transformants, around 30 transformants from each mutational type, (Matα:Δ57–70, Matα:Δ30–43, Matα:Δ47–49, Matα:Δ57–70;30–43, Matα:Δ57–70;47–49) preceding the CO-*GCSF* were directly inoculated into the BMMY medium and cultivated in a 48-well plate. A representative gel is shown for the level of G-CSF obtained with 13 different colonies obtained from the Matα:Δ57–70 construct (Fig. 5a). The data for the remaining 16 clones from this category is shown in Additional file 1: Fig. S2B. Considerable variation was detected among the different transformants. The average value of G-CSF produced in each category (from 15 such clones cultivated from each category) is shown in Fig. 5b and indicates a different trend. First, on an average, higher levels of extracellular G-CSF were produced from the codon optimized copy relative to the WT-*GCSF*. While deletion in the 57–70 amino acids increased the production by nearly 1.8-fold (Fig. 5b), deletion of the 30–43 stretch did not impact the levels of extracellular G-CSF in the same way as these did in the case of the WT-*GCSF* construct. In the double deletion, no effect was seen by the second deletion in 57–70 stretch. Deletion in the 47–49 stretch resulted in an increase in the level of extracellular G-CSF (unlike that observed with the WT-*GCSF* construct) although to not the same extent as that observed with the 57–70 deletion. Combining these two deletions did not lead to higher production of G-CSF compared to that obtained with individual deletions. The `best producer’ colonies were screened for comparison of extracellular G-CSF under uniform conditions of cultivations. Among Matα:Δ57–70 clones, Cl #s 25, 32 were chosen and a list of other clones is shown in Table 2.

### Comparative analysis of G-CSF production among the *P. pastoris* transformants containing different truncated pro-secretory sequences fused to the CO-*GCSF* gene

**Fig. 6:**
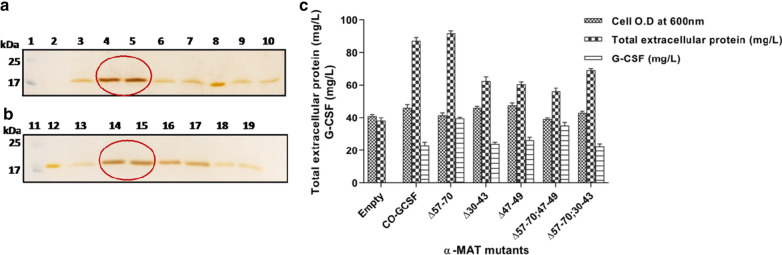
**a** SDS-PAGE analysis of extracellular G-CSF produced by transformants containing single deletions in the pro-secretory sequence fused to the CO-*GCSF* gene; Equal volume (10 μl) of the culture filtrate was loaded after 120 h of cultivation in a 500 ml Erlenmeyer flask. Lane 1: Molecular weight ladder; Lane 2; empty vector; Lane 3: Matα:WT fused to the CO-*GCSF* gene; Lanes 4 & 5: Matα:Δ57–70, Cl #s 25, 32; Lanes 6 & 7: Matα:Δ30–43, Cl# 12, 32; Lane 8: Filgrastim (1.5 μg); Lanes 9 & 10: Matα:Δ47–49, Cl #s 44, 45. **b** SDS-PAGE analysis of the G-CSF produced by transformants containing double deletions in the pro-secretory sequence fused to the CO-*GCSF* gene; Lane 11: Molecular weight ladder; Lane 12; Filgrastim (1.5 μg); Lane 13: Matα:WT fused to the CO-*GCSF* gene; Lanes 14 &15: Matα:Δ57–70, Cl# 25, 32; Lanes 16 &17: Matα:Δ57–70;47–49, Cl# 17,18; Lanes 18 & 19: Matα:Δ57–70;30–43, Cl# 23, 33. **c** Cell O.D, total extracellular protein and G-CSF titre at 120 h post-methanol induction. The units for cell O.D. are the same as shown on the Y-axis

Comparative analysis of production of G-CSF was carried out under uniform conditions of cultivation as done for the constructs with the WT-*GCSF* construct**.** SDS-PAGE analysis followed by silver staining confirmed the different levels of production of G-CSF in different categories of mutants (Fig. 6a, b). Based on the data collected at shake flask level from different categories of mutants, it was observed that the cell O.D was between 40–50 and the final pH values were between 6.0 and 6.2 in all selected transformants. Total extracellular protein varied from 60 to 100 mg/L among different transformants (Fig. 6c).

Deletion of the 57–70 amino acids in the pro-peptide resulted in production of 39.4 mg/L of G-CSF as compared to that under the native signal sequence which was ~ 23 mg/L (Fig. 6c). The deletion of the 30–43 segment resulted in G-CSF production to 24 mg/L, a value similar to that obtained with the native signal sequence. This data was different from that obtained with 30–43 deletion, when fused to WT-*GCSF*, where no levels of G-CSF were detected indicating important role of this stretch in regulation of secretion. Here, codon optimization played an important role in that better translational rates facilitated extracellular production of G-CSF. The Δ47–49 deletion also did not affect extracellular production unlike in the WT-*GCSF* construct. Thus, improved translational rates (as in the CO-*GCSF* construct) could act as a buffer and facilitated secretion.

When the deletion of 30–43 amino acids was coupled with the deletion of 57–70 amino acids, no additional increase was obtained in production of G-CSF. Further, stimulation in extracellular G-CSF levels occurred when the Δ47–49 deletion was coupled with the 57–70 deletion. However, no additive effect was seen in this double deletion construct.

### Secondary structures and predicted 3D model of truncated secretory signal sequence

Secondary structure of different truncated α-MAT versions was predicted using I-TASSER (Additional file 1: Fig. S3) to understand the relationship between structural changes and its impact on the secretion of G-CSF. On the basis of these structures, the percentage of helix, beta strands and the coil region was calculated and the results are shown in Additional file 1: Table S1. As seen, the deletion of the 57–70 region resulted in a major distortion of the beta strand that increased the overall coil percentage from 47.1 to 57.5 thereby promoting the flexibility of the loop. On the other hand, deletion in the 30–43 region of the pro-peptide resulted in an increase in % helix to 49.3% and was very similar to that observed with the double deletion construct of 30–43 and 57–70. With deletion in the 47–49, no major change was seen in the content of helix but increase in the coil region was observed in the double deletion construct with 57–70.

**Fig. 7:**
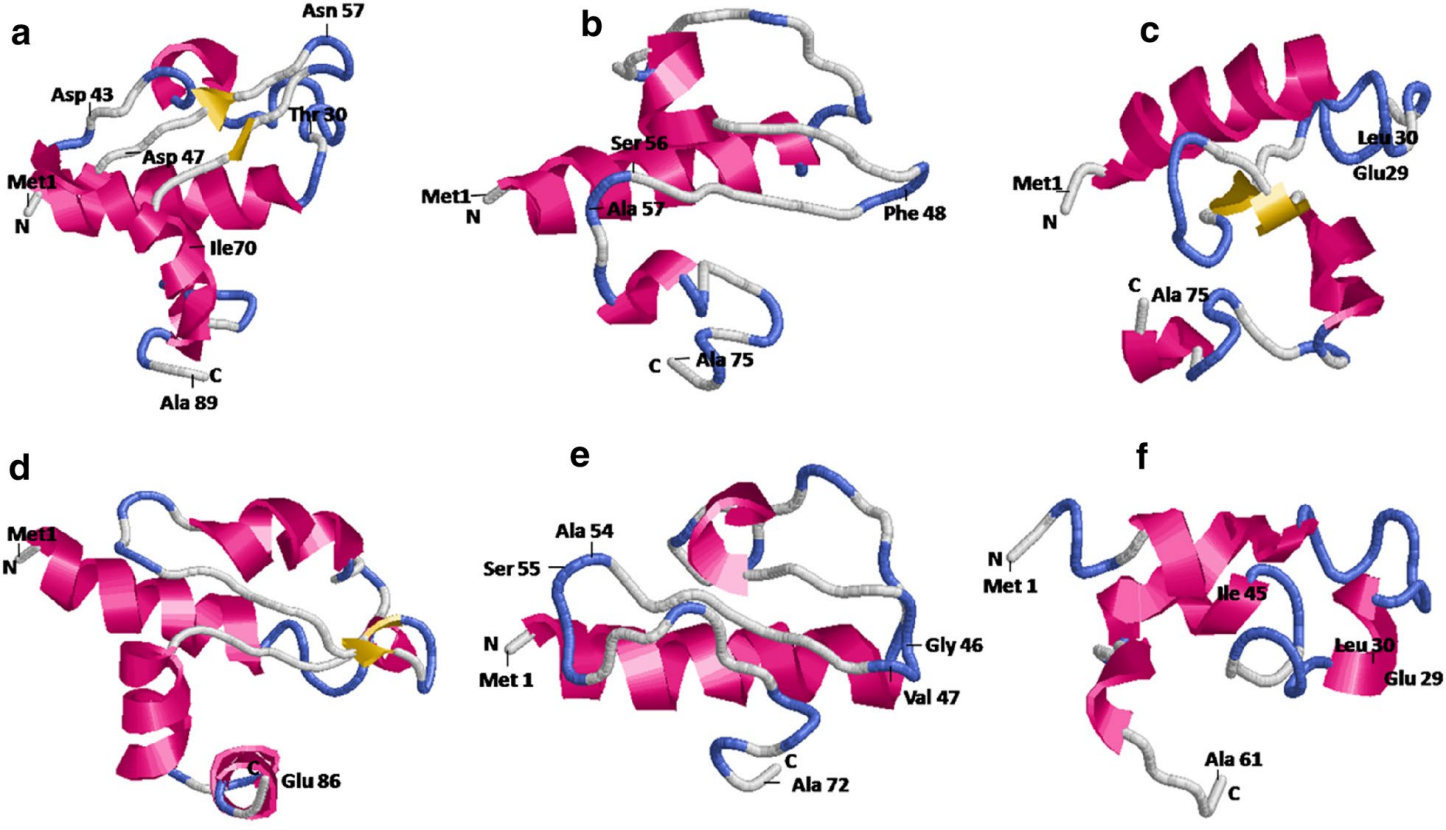
Predicted 3-D models of **a** wild type α- mating factor with 89 amino acids **b** Matα:Δ57–70 with 75 amino acids **c** Matα:Δ30–43 with 75 amino acids **d** Matα:Δ47–49 with 86 amino acids **e** Matα:Δ57–70;47–49 with 72 amino acids and **f** Matα:Δ57–70;30–43 with 61 amino acids

Three-dimensional model of the truncated secretion signal peptide was generated using I-TASSER. The model predicted the structural changes associated with the deletion of specific amino acid residues in the secretory signal peptide and helped in understanding how it may impact the transport of the cargo protein to the lumen of the ER. The wild type α-MAT consists of four α-helices and three β-strands as seen in Fig. 7a. Deletion of the amino acid residues from 57–70 led to a distortion in the 3rd α-helix and the β-strand (Fig. 7b), allowing lengthening of the loop. This is likely to allow increased interaction with the cargo protein and assist the secretion process. On the other hand, deletion of the amino acid residues from 30–43 (Fig. 7c) and 47–49 (Fig. 7d) led to major disruption in the 2nd α-helix and shortening of the loop respectively indicating constrained interaction with the cargo protein. This resulted in dampening of the secretion in case of the WT-*GCSF* construct. However, such changes did not impact the protein emerging from a CO-*GCSF* copy of the gene. Combination of these deletions (Δ57–70; 47–49) led to a distortion of the 3rd α -helix and shortening of the loop (Fig. 7e) whereas deletion of the 57–70 and 30–43 amino acids led to distortion of the 2nd and the 3rd α -helix of the α-MAT (Fig. 7f) peptide.

## Discussion

*P. pastoris* is a well-defined eukaryotic expression system and has been extensively used for the production of many heterologous proteins. Many industrially important proteins have been reported to reach high production levels (15 g/L), yet there are few proteins that are produced at mg/L levels, including G-CSF. Since there are many advantages of using this system as a host for production of human therapeutics, strategies need to be developed that ensure increase in the yield of the foreign proteins.

Out of the many secretory signal sequences available for extracellular production of heterologous proteins in the *P. pastoris* system, the α-MAT pre-pro signal peptide has been used widely and remains one of the most successfully used sequences. Both the pre- and the pro- regions are important during processing and secretion of the mature protein. The pre-peptide plays an important role in guiding the newly synthesized protein to the cavity of the ER by interacting with the signal recognition particle [33]. The pre-peptide also guides co-translational or post-translational transport of the newly synthesized protein. The role of the pro-peptide, which is hydrophobic in nature and interrupted by short stretches of charged or polar amino acids, is still not clear. It is proposed to slow down the transport of the protein to ensure proper folding [60, 61].

In this study, work was undertaken with an objective of enhancing the extracellular G-CSF production in X-33 strain of *P. pastoris.* A number of studies report on the production of G-CSF in GS115 [45–47] but the yield has been around 4–10 mg/L at shake flask level. A two- stage glycerol feeding strategy was applied at bioreactor level which resulted in production of 320 mg/L of human G-CSF [62]. Also, the same group of researchers studied the effect of non-ionic surfactants like Tween 80, Tween 20 and betaine on the production of G-CSF. It was found that the addition of non-ionic surfactant into the medium did not increase the product yield but prevented the G-CSF from aggregation. However, other groups have achieved 125-fold increase in G-CSF production after the addition of Tween 80 during shake flask studies [63]. The biological activity was 1.6-fold lower as compared to the *E. coli* derived G-CSF. In a recent study, codon optimized copy of the gene for G-CSF was expressed under the control of the *AOX1* promoter in the SMD1168 strain and this led to extracellular production of 18 mg/L G-CSF at shake flask level [48]. However, these strains are not as effective for large scale production as the X-33 strain due to their lower specific growth rate, low viability, and less transformation efficiency [20]. When the gene for G-CSF was fused with a DNA fragment encoding Domain III of HSA, it resulted in production of 250–300 mg/L of the total protein [50] with over 37% constituting G-CSF. However, obtaining a cleaved therapeutic from such fusion constructs remains a challenge. The G-CSF produced in the present study was in the X-33 host and the identity of the protein produced was established by performing MALDI/TOF analysis.

Changes in the signal sequences preceding the WT-*GCSF* and CO-*GCSF* was taken up as a novel strategy to understand the implications of their effect on extracellular production as well as to gain an understanding of how translation and secretion could be coupled. If these were unlinked, such mutations would impact G-CSF production from the CO-*GCSF* gene as much as they would from the WT-*GCSF* gene. If these were linked, one should expect to see a different response from the two sets. Four of the mutations studied in the α-MAT secretory signal (i.e. Δ57–70, Δ30–43, Δ57–70; 30–43, Δ57–70; 47–49) were the same as reported in an earlier study [41] and were chosen because of their indicated enhancement in production of horse radish peroxidase (HRP) and the fifth mutation of 47–49 deletion was a new one. One of the common observations in the *P. pastoris* system is the  variability in extracellular production of the foreign protein among the clones [56–58] from the same integration event. When a detailed genome analysis was carried out of such clones, the integration event was found to occur at different loci, and, this in turn, affected the extracellular production of the foreign protein [58, 59]. Thus, it is important to choose a large number of clones to see the effect of such mutations, which has been largely missing in the previous study [41]. In the present study, this was avoided by screening a number of colonies and looking at the average level of production in the clones arising from transformation of a plasmid containing one mutation in the α-MAT signal. The `selected producer’ clones were next chosen from each category and cultivated under uniform conditions of cultivation and the supernatant analysed for the production of G-CSF based on a gel densitometry method. Since silver staining was used in the process, no external bias was introduced which may occur due to enzyme assays or concentration of the culture supernatant. Second, it is well known that the methanol utilization phenotype affects production of the extracellular protein [64] and the Mut^s^ (methanol *slow* utilization phenotype, due to integration replacing the *AOX1* locus) variants have been shown to produce higher levels of the foreign protein in many cases. Although the chances of obtaining the Mut^s^ are small in the X-33 host, these do occur and thus the colonies selected need to be screened for the Mut phenotype. These have been reported to occur at a frequency of 1–5/20 His^+^ transformants in the *P. pastoris* GS115 strain [65] as a double crossover event is required for obtaining this phenotype. Thus, screening was carried out to ensure that the differences occurring due to deletions in the secretory signal sequences are captured accurately.

**Fig. 8:**
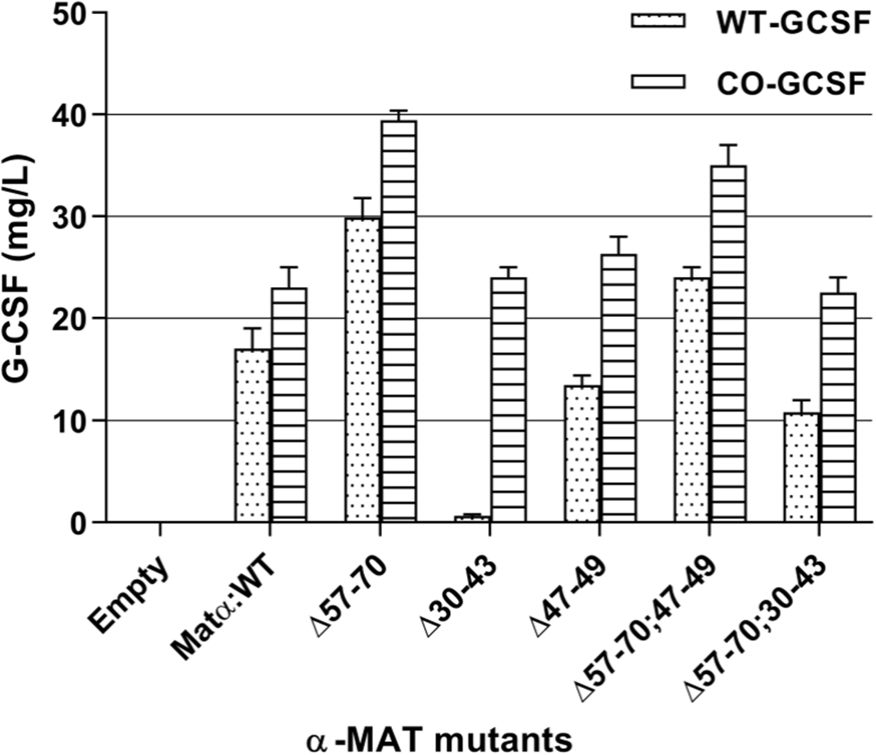
Comparative analysis of extracellular G-CSF produced in *P. pastoris* transformants containing truncated α-MAT mutants preceding either the WT-*GCSF* gene or the CO-*GCSF* gene

The effect of different deletions in the pro-region preceding the native cDNA or the codon optimized copy of G-CSF gene is summarized in Fig. 8. Several important deductions can be made. First, deletion of the 57–70 amino acid residues in the pro-peptide resulted in an ~2-fold higher G-CSF production, irrespective of whether the deletion preceded the WT- or the CO-*GCSF* construct, except that the amount produced through the codon optimized copy was more. Similar effect of deletion of the 57–70 amino acids was reported [41] on production of HRP. Second, deletion of the 30–43 amino acids in the pro-peptide fused with the WT-*GCSF* completely diminished extracellular production of G-CSF which was different from the results obtained in a previous study where this deletion caused an increase in the level of HRP. It was also different from the observations made with this deletion, next to the CO-*GCSF* gene, where no effect was seen on the extracellular G-CSF production. Thus, higher rates of translation compensated the deleterious effect of Δ30–43. The effect of this deletion could be partly compensated for by deletion of 57–70 region in the WT-*GCSF* construct. Hence, the present study indicates an important role of the 30–43 amino acid stretch in the α-MAT signal in the secretion of protein. Third, the present study also indicates the importance of the 47–49 segment and this effect was revealed in the double deletion construct (Δ57–70; 47–49) where the values of extracellular G-CSF were lower when compared to the values obtained with the Δ57–70 alone. The data also suggests that irrespective of the amount of G-CSF produced, the processing of the leader peptide was similar and no stress was observed on the secretion processes. In general, stress on the secretory pathway is reported to be associated with lowering of cell growth, among other factors [66], and in all categories of mutants (derived with either the WT-*GCSF* or the CO-*GCSF)*, the cell density was nearly the same. As seen in Figs. 4c and 6c, the cell density (by way of measuring cell O.D._600_) varied between 40 and 50 for all constructs indicating that there was no stress on the cellular growth.

In order to understand the role played by the tertiary structure of the leader peptide in binding to the cargo protein and regulate secretion, the 3-D models were built of the pro-region in different deletion mutants. The 57–70 amino acids of the secretory signal form a major part of the 3rd α-helix. Deletion of these amino acid residues leads to a distortion in this α-helix, allowing flexibility of the loop now created which increased its interaction with the cargo protein and thus allowed higher secretion. Thus, mutations that increase this flexibility would favour the secretion while mutations which constrained this loop would restrain the interactions and hence would decrease the secretion of the cargo protein. On the other hand, the 30–43 amino acids form a part of the 2nd α-helix which appears to be important for transport. The involvement of this second helix has not been reported earlier. Thus, apart from binding to the 3rd helix (which regulates the transport), the cargo protein must also bind to the 2nd α-helix. If the rates of translation are high, binding to the loop region is enough (created on account of deletion of 57–70 region) to propel the protein to ER. The model indicated that the 47–49 amino acids formed a part of the loop region and while its removal did not appear to impact the secretion but in the event of its coupling with 57–70 deletion, the loop size was considerably reduced and this impacted the secretion. These double deletions did not achieve the same level of extracellular production as that achieved by the 57–70 deletion alone (Fig. 8). Hence, this region was also concluded to be important. Whether the final conformation of the cargo protein (α-helical, β-sheet, α/β etc.) also plays a role is still not known although majority of the α-helical proteins are known to be secreted in high amounts. Several other studies also indicate the prominent role played by the α-MAT sequences in the secretion process. For instance, a mutant library of the α-MAT pre-pro signal peptide was generated by error prone PCR mutagenesis and in one of the isolated mutants, secretion of the single chain antibody was increased by 16-fold over the wild type construct [67] while in most others, increased extracellular production of the antibody was observed. On sequencing eight of the best leader sequences, a definite trend was noticed in that the 22nd Val, in the WT α-MAT, was changed to an Ala. Also, the hydrophobic LLFI motif (at the position of 63–66 amino acids) was mutated to more polar amino acids indicating that even point mutations can significantly change the properties of the leader in its ability to transport the cargo protein. However, no data was provided on the structural changes that may accompany these point mutations. Another study reported enhanced secretion of phytase protein by 7-fold which could be accomplished after codon optimization of the α-MAT secretory signal sequences [43]. Fusion of the pro-region of the α-MAT with signal peptide of mouse salivary α-amylase [68] or the Ost1 signal of *S. cerevisiae* [44] has been reported to result in increased expression of *Rhizopus oryzae* glucoamylase by 3.6-fold or of red fluorescent protein E2-Crimson by 20-fold respectively. The fusion with the Ost1 signal peptide, wherein co-translational transport of the cargo protein occurred, also worked favourably (10-fold increase) for lipase BTL2 protein. In addition to the α-MAT, several indigenous signal peptides (such as those of cell wall protein Scw, daughter cell-specific secreted endoglucanase like protein Dse and exoglycanase Exg) are also being investigated [69] and can hold promise for increasing production of foreign proteins in the *P. pastoris* system.

To reconfirm if the modifications in the signal sequence did not affect the intracellular level of G-CSF in different category of mutants, the cell-free extract was loaded and resolved on the SDS-PAGE gel. However, the levels of G-CSF were comparable to that obtained when the WT α-MAT secretory signal was used. While these results indicate an important role of the 2nd, 3rd helix and the intervening loop, some other studies have recently concluded [70] that both the N and the C-terminus of the α-MAT pro-peptide also need to be presented in a specific orientation for interaction with the cellular secretion machinery and for efficient protein secretion.

## Conclusions

The fine tuning between production and secretion of G-CSF was revealed by observing the response of the WT-*GCSF* and the CO-*GCSF* gene to deletions in different parts of the pro-region of the α-MAT secretory signal sequence. An enhancement in extracellular production of G-CSF was achieved (~ 39.4 mg/L) at shake flask level by using a codon optimized copy of the gene and stitching it to specific deletion (Δ57–70) in the pro-region of the α-MAT secretory signal. Two important findings emerged from these detailed studies: first, an increase in the coiled region (or presence of unstructured peptide) provides for greater flexibility in the pro-peptide and facilitates export of the cargo protein; second, an increase in the content of helix appears to regulate export strongly for the newly emerging peptide from the WT-*GCSF* construct but not from a rapidly synthesized peptide from the CO-*GCSF* construct. Thus, while some tertiary structure is required for binding to the cargo protein, presence of helical regions restricts extracellular transport. While individual deletions increased the production from CO-*GCSF* copy, no cumulative effect was seen in the double deletion constructs indicating that shortening of the loop is also not desirable. Such fine tuning can serve as a guide while producing foreign proteins in the *P. pastoris* system. The data also suggested that the engineering of the leader peptide can be a powerful approach towards enhancing productivity of a foreign protein. Using an engineered host (that contain other markers that facilitate formation of disulfide bonds, produce specific transcription factors) and applying specific cultivation strategies at bioreactor level can further lead to improved productivities.

## Supplementary information

**Figure S1. **Screening of methanol utilization phenotype of Matα:Δ57-70 by PCR. **Figure S2.** SDS-PAGE analysis of the extracellular G-CSF produced by other clones containing the truncated α-MAT (deletion of amino acids 57-70 or Δ57-70) fused to (A) the WT-*GCSF* or (B) to the CO-*GCSF* gene. **Figure S3.** Predicted secondary structure of the truncated Matα mutants. **Table S1. **Helix, strand and coil percentage of the truncated α-MAT.

## Data Availability

All data generated or analysed during this study are included in this published article [and its Additional files].
