# Supplementary material for: Differential role of segments of α-mating factor secretion signal in Pichia pastoris towards granulocyte colony-stimulating factor emerging from a wild type or codon optimized copy of the gene
Source: Microb Cell Fact. 2020 Oct 29;19:199. doi: 10.1186/s12934-020-01460-8 (PMC7597063; doi:10.1186/s12934-020-01460-8)
Supplement: Supplementary file 1 — Figure S1. Screening of methanol utilization phenotype of Matα:Δ57-70 by PCR. Figure S2. SDS-PAGE analysis of the extracellular G-CSF produced by other clones containing the truncated α-MAT (deletion of amino acids 57-70 or Δ57-70) fused to (A) the WT-GCSF or (B) to the CO-GCSF gene. Figure S3. Predicted secondary structure of the truncated Matα mutants. Table S1. Helix, strand and coil percentage of the truncated α-MAT. [file 12934_2020_1460_MOESM1_ESM.docx]

**Figures**

**
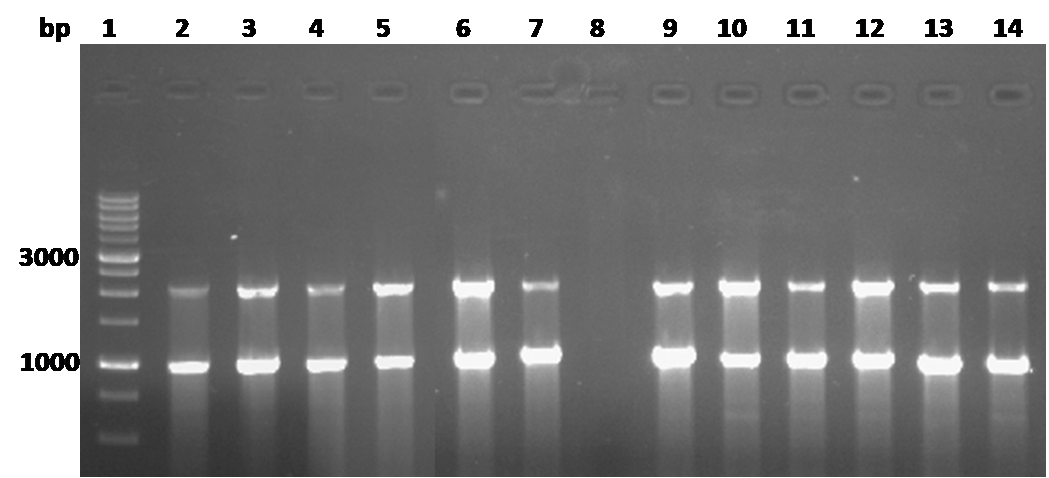
**

**Fig. S1** Screening of the methanol utilization phenotype of Matα:△57-70 by PCR. Lane 1: Molecular weight ladder; Lanes 2 to 14: PCR amplified product with upper band (2.2 kb) and lower band (1.2 kb) from several randomly picked colonies.

1. B.


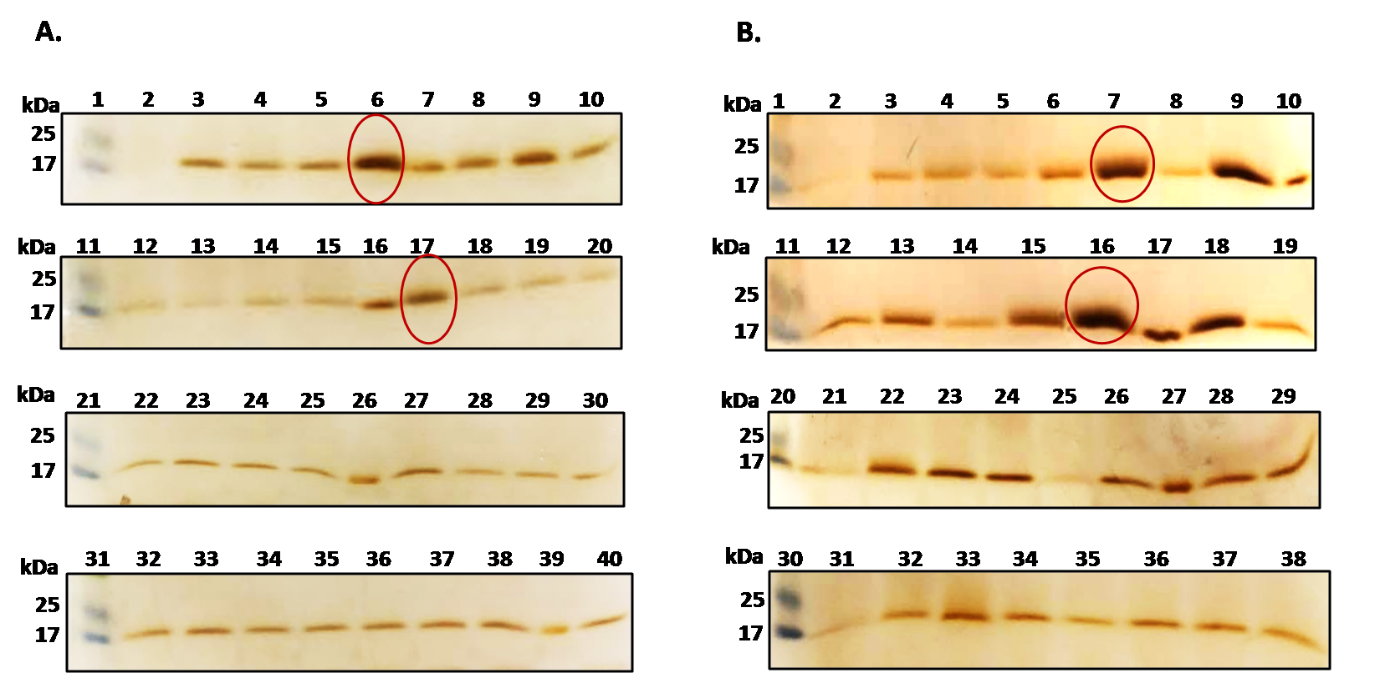


**Fig. S2 (A)** SDS-PAGE analysis of extracellular G-CSF produced by other 15 clones containing the truncated α-MAT (deletion of amino acids 57-70 or Δ57-70) fused to the WT-*GCSF*. Equal volumes (25 μl) were loaded after 72 h of cultivation in the BMMY medium in a 48-well plate. Lanes 21 & 31: Mol wt markers; Lanes 22, 23, 24, 25, 27, 28, 29, 30: Cl #s 20 to 27; Lane 26: Standard Filgrastim (0.5 μg); Lanes 32-38, 40: Cl #s 28 to 35; Lane 39: Standard Filgrastim (0.3μg)

**(B)** SDS-PAGE analysis of extracellular G-CSF produced by other 15 clones containing the truncated α-MAT (deletion of amino acids 57-70 or Δ57-70) fused to the CO-*GCSF* in BMMY medium. Equal volumes (25 μl) were loaded after 72 h of cultivation in the BMMY medium in a 48-well plate. Lanes 20, 30: Mol wt markers; Lanes 21, 22, 23, 24, 25, 26, 28, 29: Cl #s 36 to 43; Lane 27: Standard Filgrastim (1.8 μg); Lanes 31 to 38: Cl #s 44 to 51.

**Fig. S3** Predicted secondary structure of truncated Matα mutants **(a)** Wild type α-mating factor (Matα:Wt) **(b)** Matα:Δ57-70 **(c)** Matα:Δ30-43 **(d)** Matα:Δ47-49 **(e)** Matα:Δ57-70;47-49 **(f)** Matα:Δ57-70;30-43. Helix (H), Strand (S), Coil (C)

**Table S1 Helix, strand and coil percentage of truncated α-mating factor**

| **WT/Mutant type** | **% helix** | **% strand** | **% coil** |
| --- | --- | --- | --- |
| Matα:Wt | 33.7% | 19.1% | 47.1% |
| Matα:△57-70 | 33.3% | 9.3% | 57.3% |
| Matα:△30-43 | 49.3% | 16% | 49.3% |
| Matα:△47-49 | 34.8% | 19.7% | 45.3% |
| Matα:△57-70;30-43 | 44.2% | 8% | 47.5% |
| Matα:△57-70;47-49 | 33.3% | 13.8% | 52.7% |
